# Common *ADRB2* Haplotypes Derived from 26 Polymorphic Sites Direct β_2_-Adrenergic Receptor Expression and Regulation Phenotypes

**DOI:** 10.1371/journal.pone.0011819

**Published:** 2010-07-29

**Authors:** Alfredo Panebra, Wayne C. Wang, Molly M. Malone, Demar R. G. Pitter, Scott T. Weiss, Gregory A. Hawkins, Stephen B. Liggett

**Affiliations:** 1 Cardiopulmonary Genomics Program, University of Maryland, Baltimore, Maryland, United States of America; 2 Channing Laboratory, Department of Medicine, Brigham and Women's Hospital, Harvard Medical School, Boston, Massachusetts, United States of America; 3 Wake Forest University, Winston-Salem, North Carolina, United States of America; University of Cincinnati, United States of America

## Abstract

**Background:**

The β_2_-adrenergic receptor (β_2_AR) is expressed on numerous cell-types including airway smooth muscle cells and cardiomyocytes. Drugs (agonists or antagonists) acting at these receptors for treatment of asthma, chronic obstructive pulmonary disease, and heart failure show substantial interindividual variability in response. The *ADRB2* gene is polymorphic in noncoding and coding regions, but virtually all *ADRB2* association studies have utilized the two common nonsynonymous coding SNPs, often reaching discrepant conclusions.

**Methodology/Principal Findings:**

We constructed the 8 common *ADRB2* haplotypes derived from 26 polymorphisms in the promoter, 5′UTR, coding, and 3′UTR of the intronless *ADRB2* gene. These were cloned into an expression construct lacking a vector-based promoter, so that β_2_AR expression was driven by its promoter, and steady state expression could be modified by polymorphisms throughout *ADRB2* within a haplotype. “Whole-gene” transfections were performed with COS-7 cells and revealed 4 haplotypes with increased cell surface β_2_AR protein expression compared to the others. Agonist-promoted downregulation of β_2_AR protein expression was also haplotype-dependent, and was found to be increased for 2 haplotypes. A phylogenetic tree of the haplotypes was derived and annotated by cellular phenotypes, revealing a pattern potentially driven by expression.

**Conclusions/Significance:**

Thus for obstructive lung disease, the initial bronchodilator response from intermittent administration of β-agonist may be influenced by certain β_2_AR haplotypes (expression phenotypes), while other haplotypes may influence tachyphylaxis during the response to chronic therapy (downregulation phenotypes). An ideal clinical outcome of high expression and less downregulation was found for two haplotypes. Haplotypes may also affect heart failure antagonist therapy, where β_2_AR increase inotropy and are anti-apoptotic. The haplotype-specific expression and regulation phenotypes found in this transfection-based system suggest that the density of genetic information in the form of these haplotypes, or haplotype-clusters with similar phenotypes can potentially provide greater discrimination of phenotype in human disease and pharmacogenomic association studies.

## Introduction

The β_2_-adrenergic receptor (β_2_AR) is a member of the G-protein coupled receptor superfamily (GPCR), and is expressed on virtually all cell-types [1]. The catecholamines epinephrine and to a lesser extent norepinephrine are the endogenous agonists which activate these receptors as part of the sympathetic nervous system. Of particular interest for therapeutic purposes have been β_2_AR expressed on smooth muscle cells of the airway which promote bronchodilatation and those expressed on cardiomyocytes which promote cardiac contractility. β-agonists acting on airway smooth muscle β_2_AR, and non-selective β-blockers acting on β_2_- and β_1_AR on cardiomyocytes, are standard treatments of obstructive lung disease and heart failure, respectively.

The β_2_AR gene (*ADRB2*), which is localized to 5q31-32, is intronless and encodes a protein of 413 amino acids that has a seven transmembrane topology typical of the superfamily. The amino-terminus is extracellular while the carboxy-terminus is intracellular. Ligand binding occurs in a “pocket” formed by residues of the transmembrane domains. The substantial interindividual variability in the responses to β-agonists [2] and antagonists [3] has prompted examination of the gene for common polymorphisms. Our identification [4] of coding polymorphisms of the β_2_AR in 1993 represented the first report of discreet nonsynonymous polymorphisms of any GPCR and prompted a host of subsequent clinical association studies [5], [6]. The two common non-synonymous single nucleotide polymorphisms (SNPs, Table 1) are at nucleic acid 46 resulting in amino acid position 16 being either Arg or Gly, and at nucleic acid 79 resulting in amino acid position 27 being Glu or Gln [4]. These polymorphisms are localized to the extracellular amino-terminus, and have a subtle effect on agonist-promoted downregulation of receptor expression when studied in a cell-based system where transfections of vectors containing only the open reading frames were carried out [7]. Virtually all association studies for pharmacogenomic effects have utilized these coding polymorphisms with variable or inconsistent results (reviewed in [5], [8]). These inconsistent associations have prompted examination of the promoter, 5′UTR, and 3′UTR of *ADRB2* for additional polymorphisms. Initial studies by our group indicated multiple polymorphisms in these other regions, many of which were not in significant linkage disequilibrium with the coding polymorphisms [9]–[11]. Thus there is the potential for a more precise *ADRB2* genetic signature for association studies, which may be particularly important if these other polymorphisms affect receptor expression or agonist mediated regulation of the receptor. Of interest is the potential for interaction between polymorphisms, such as promoter polymorphisms that alter transcription and coding polymorphisms that alter protein stability, so that the net effect could be a composite of multiple variants on the phenotype. Based on our previous studies of this intronless gene, there appear to be 17 common SNPs in the 5′ upstream region (3500 bases 5′ of the initiator ATG), 7 coding SNPs (3 nonsynonymous), and one SNP and a variable poly-C tract in the 3′UTR (Table 1). In subjects of European or African ancestry, these are found to be arranged into eight haplotypes with frequencies of >0.05 in at least one racial group [11]. These common haplotypes have been named based on our initial haplotypes that only considered the 5′-flanking and coding regions [9], and are denoted I-1, II-1, IV-1, IV-2, IV-3, IV-4, VI-1 and VI-2 (Table 1). The cellular phenotypes of these combinations of SNPs, based on the common haplotypes, have not been determined. Because of the intronless nature of *ADRB2*, “whole-gene” transfections are possible and provide an opportunity to ascertain the effects of the combination of SNPs, as they appear in nature, on cellular phenotypes. The current work utilizes this approach, where we identify phenotypes which have not been previously appreciated by studying individual SNPs or limited groups of SNPs.

**Table 1:** Localization of the common polymorphisms of *ADRB2*.

	−3459	−3291	−3287	−3251	−3159	−2633	−2387	−2274	−1818	−1531	−1429	−1343	−1023	−654	−468	−367	−20	46	79	252	523	1053	1205	1239	1266–1275	1276
	T/C	C/T	T/A	A/C	T/C	T/C	C/T	C/T	A/T	C/T	T/A	A/G	A/G	G/A	C/G	T/C	T/C	A/G	C/G	G/A	C/A	G/C	A/C	A/G	C	G/C
**Haplotype**																										
**I-1**	T	C	T	A	T	T	C	C	A	C	T	A	A	G	C	T	T	A	C	G	C	G	A	A	9C	G
**II-1**	C	C	T	A	T	T	C	C	A	C	T	A	A	G	G	C	C	G	G	G	C	G	A	G	7C	C
**IV-1**	T	T	A	C	T	C	T	T	T	C	T	G	G	A	C	T	T	A	C	G	C	G	A	G	8C	C
**IV-2**	T	T	A	C	T	C	T	T	T	C	T	G	G	A	C	T	T	A	C	G	C	G	A	G	9C	C
**IV-3**	T	T	A	C	T	C	T	T	T	C	T	G	G	A	C	T	T	A	C	G	C	C	A	A	10C	G
**IV-4**	T	T	A	C	T	C	T	T	T	C	T	G	G	A	C	T	T	A	C	G	C	G	A	G	7C	C
**VI-1**	T	T	A	C	C	C	T	T	A	T	A	G	G	G	C	T	T	G	C	A	A	C	C	A	9C	G
**VI-2**	T	T	A	C	T	C	T	T	A	T	A	G	G	G	C	T	T	G	C	A	A	C	C	A	9C	G

Shown are the common haplotypes (allele frequency>0.05 in either white subjects of European descent or black subjects of African descent. Haplotype names, and their prevalence in these populations, are given in references [8] and [10].

## Results and Discussion

**Figure 1:**
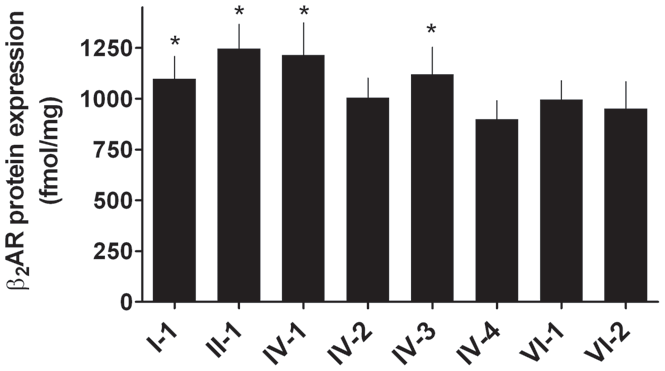
β_2_AR protein expression phenotypes of the common *ADRB2* haplotypes. COS-7 cells were transfected with constructs representing the β_2_AR haplotypes shown in Table 1. Receptor protein expression was determined by quantitative radioligand binding. Results are from 7 experiments. *, p<0.05 vs. the other haplotypes.

**Figure 2:**
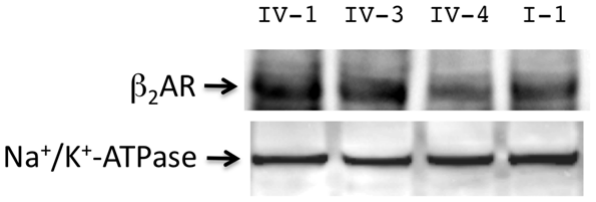
Western blots confirm the radioligand binding method for ranking expression. Monoclonal antibody to a non-polymorphic region of the β_2_AR protein was utilized to confirm the radioligand binding method for ranking expression phenotypes of the β_2_AR haplotypes. Shown is a single experiment revealing higher expression of haplotypes IV-1 and IV-3 compared to IV-4 and VI-2 β_2_AR protein at the expected molecular weight (∼79 kDa). These results are consistent with those from radioligand binding of Figure 1. The control antibody was to Na^+^/K^+^-ATPase, a cell membrane protein.

The constructs utilized for transfection consisted of 5,580 bp of *ADRB2* (−3685 to +1895) which represents contiguous promoter, 5′UTR, coding and 3′UTR sequence up to the poly-A termination site. Site-directed mutagenesis and digestion-ligation reactions were utilized to construct the eight haplotypes, which were verified by sequencing (Table 1). The final constructs were cloned into a modification of the pcDNA3.1(+) expression vector in which the CMV promoter was removed (denoted pcDNA3.1(+)/ΔCMV, see Methods). Thus expression of the β_2_AR was driven by its own promoter, and influences of promoter, 5′UTR, coding and 3′UTR polymorphisms, in their appropriate context (i.e., the eight haplotypes) could be ascertained. Studies were performed in transfected COS-7 cells. Two major phenotypes were considered: baseline β_2_AR protein expression, and, agonist-promoted downregulation of receptor protein expression. β_2_AR expression was ascertained using a highly quantitative ^125^I-cyanopindolol (^125^I-CYP) radioligand binding assay. In the absence of transfection, such binding revealed <10 fmol/mg expression in COS-7 cells, while transfection with the whole-gene vectors provided for ∼1,000 fmol/mg human β_2_AR expression. The results from these transfection studies are shown in Figure 1. Haplotypes I-1, II-1, IV-1 and IV-3 had higher expression levels than the other four haplotypes. To verify the radioligand binding method for ranking expression by haplotypes, Western blots were performed as shown in Figure 2 using a monoclonal antibody directed to a non-polymorphic region of the receptor protein. As indicated, haplotypes IV-1 and IV-3 had greater immunoreactivity at the expected molecular weight compared to haplotypes IV-4 and VI-2, consistent with the results from radioligand binding.

**Figure 3:**
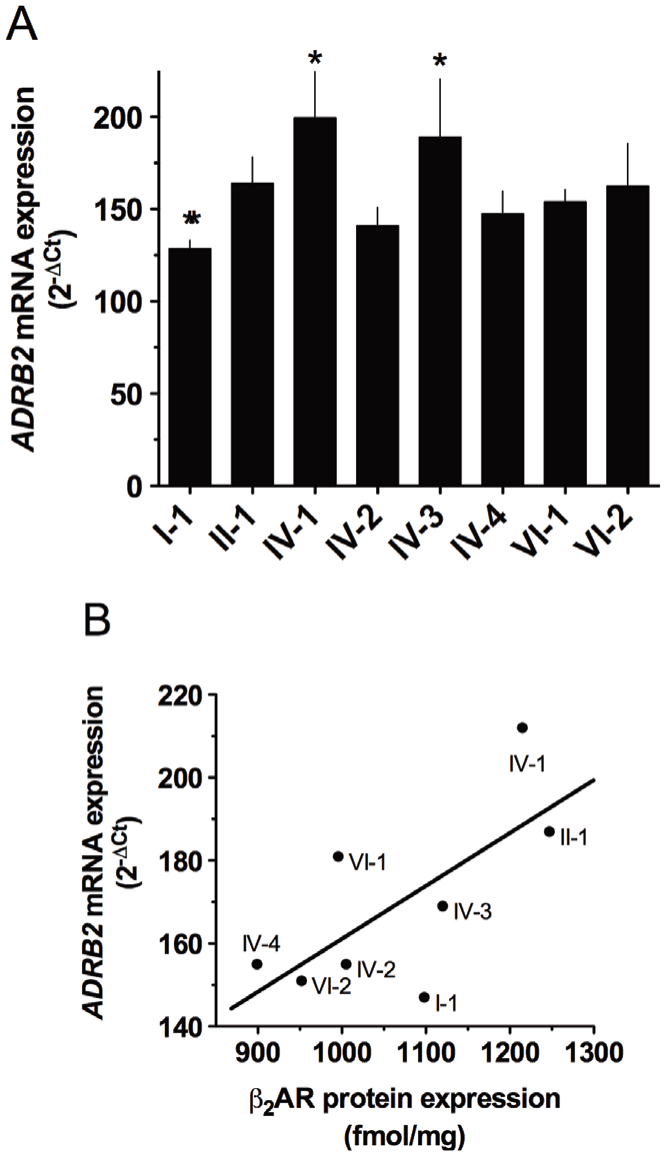
*ADRB2* mRNA levels of the transfected β_2_AR haplotypes. (A) mRNA levels for haplotypes IV-1 and IV-3 are higher, and I-1 lower, than levels of the other haplotypes. Results are from 6 experiments. *, p<0.05 vs. the other haplotypes. (B) relationship between *ADRB2* mRNA levels and β_2_AR protein expression. The r^2^ for this relationship was 0.47. Two haplotypes (VI-1 and I-1) are greater than 1 standard deviation from the mean β_2_AR/*ADRB2* ratio.

The *ADRB2* mRNA expression, ascertained by quantitative RT-PCR [12], did not necessarily mirror β_2_AR protein expression for each haplotype (Figure 3A). The relationship between protein expression and mRNA (essentially showing the ratio for each haplotype) is shown in Figure 3B. As shown, there is a trend towards a statistically significant overall correlation between *ADRB2* mRNA and β_2_AR protein (r^2^ = 0.47, p = 0.06). However, when formally tested two haplotypes (I-1 and VI-1) had β_2_AR/*ADRB2* ratios that differed by 1 standard deviation from the mean ratio. Indeed, removal of either of these outliers increases the r^2^ value to ∼0.60 with significance at p = 0.03. The basis for this difference in relative levels of mRNA vs. protein expression is not readily apparent by inspection of haplotype sequence. Haplotype VI-1 differs from its closest haplotype (VI-2, a haplotype that is consistent with the regression line) by a single nucleotide within the 5′-flanking region at position −3159 (C or T). On the other hand, VI-1 differs from the regression-conforming haplotype II-1 by 25 SNPs. A similar analysis with I-1 reveals multiple differences in sequence between it and all other seven haplotypes. Taken together, these results further support the notion that, for most haplotypes, multiple SNPs are likely interacting to ultimately establish phenotype.

**Figure 4:**
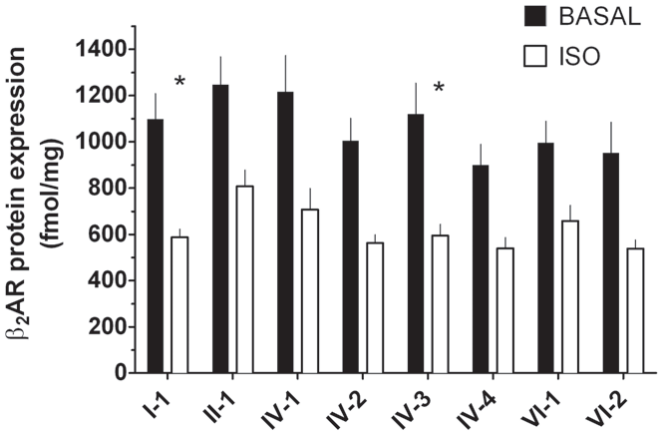
Agonist-promoted downregulation phenotypes of the common *ADRB2* haplotypes. Transfected COS-7 cells were exposed to media alone or media with 10 µM isoproterenol for 48 hours. β_2_AR protein expression was determined by quantitative radioligand binding. The results are from 7 experiments. *, % downregulation from the untreated state differs at p<0.05 vs. all other haplotypes.

**Figure 5:**
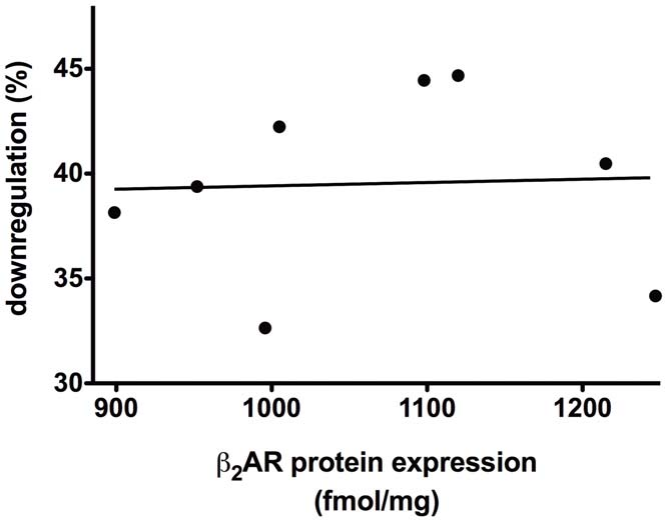
Lack of a relationship between baseline β_2_AR expression and the % downregulation. The agonist-promoted downregulation, expressed as a percentage, was plotted against baseline β_2_AR protein expression. There was no relationship (r^2^ = 0.001, p>0.9) between the initial expression of β_2_AR and the extent of downregulation. Results are from the 7 experiments of Figure 4.

We next assessed the agonist-promoted downregulation phenotype, which may be relevant to tachyphylaxis during prolonged exogenous or endogenous agonist exposure [13]. Eighteen hours after transfection, cells were exposed to vehicle (0.1 mM ascorbic acid) or the agonist isoproterenol (10 µM) for 48 hours, washed, and cell membranes prepared. ^125^I-CYP radioligand binding was performed, and the results are shown in Figure 4 compared to untreated cells. Two haplotypes (I-1 and IV-3) showed greater extents of agonist-promoted downregulation compared to the other five. One potential concern regarding defining the downregulation phenotype based on a percentage of the untreated (baseline) expression is a confoundment from differences in baseline expression. However, we found no evidence for any relationship between baseline levels of expression and the % downregulation (Figure 5).

**Figure 6:**
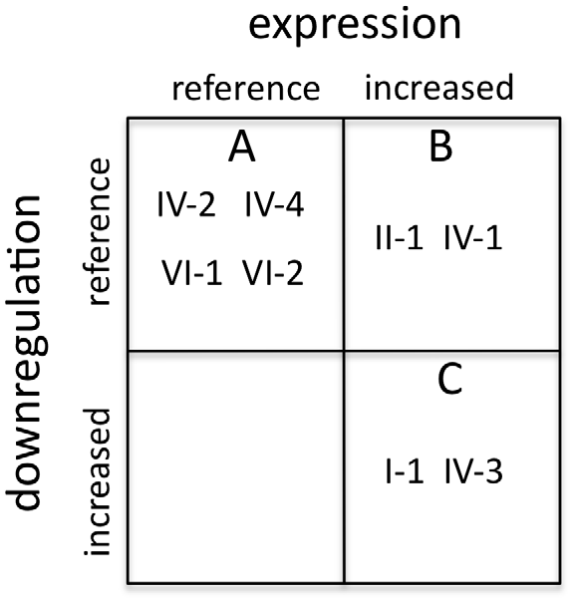
Stratification matrix. The phenotypes derived from the *in vitro* cell-based studies are stratified in a 2×2 matrix according to baseline β_2_AR expression and the extent of agonist-promoted downregulation. Three bins were populated as shown and are denoted A, B, C for clarity.

In terms of haplotypes predicting clinical responsiveness to β-agonists in asthma, our results suggest that these phenotypes are complex and can segregate as shown in Figure 6. For purposes of this grouping, expression is defined as “reference” or “increased,” and agonist-promoted downregulation as “reference” or “increased.” As shown, then, the haplotypes segregate into three groups: reference expression and downregulation, increased expression with reference downregulation, and increased expression with increased downregulation. These are designated as groups A (haplotypes IV-2, IV-4, VI-1 and VI-2), B (haplotypes II-1 and IV-1) and C (haplotypes I-1 and IV-3). Using group A as the reference, patients in group C would be predicted to have a high initial bronchodilating response to β-agonist, but would experience the greatest tachyphylaxis to chronic administration. On the other hand, patients in group B would also have a high initial response, but would experience less tachyphylaxis, so the overall best clinical response would be with these patients, which have haplotypes II-1 and IV-1. Although no studies have utilized these full haplotypes, there are some that have assessed potential associations between limited haplotypes, genotype combinations, or individual SNPs and β-agonist phenotypes in asthma [5], which can be interpreted to conform to the dual phenotypic groups of Figure 6. However, each study showed variability within the limited genetically-defined groups, consistent with additional genetic variations potentially represented by the extended haplotypes, contributing to phenotypic noise.

**Figure 7:**
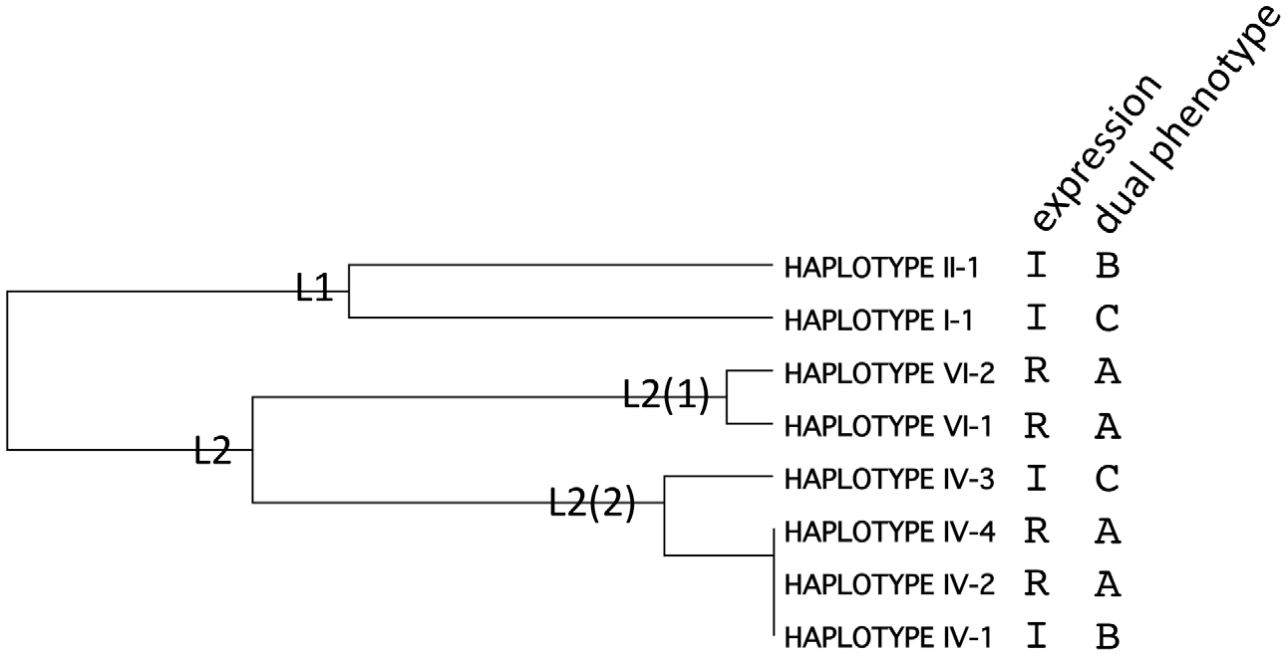
Phylogenetic tree of β_2_AR haplotypes with phenotypic annotation. Shown is a phylogenetic tree constructed as indicated in Methods with the 8 β_2_AR haplotype sequences. Shown are the expression phenotypes (I, increased; R, reference) and the dual phenotypes that incorporate expression and downregulation (denoted A, B, C from Figure 6). L1 and L2 represent the two ancestral lineages, while L2(1) and L2(2) are lineages from L2.

A phylogenetic analysis of the β_2_AR haplotypes was undertaken with an overlay of the expression phenotypes, and the dual phenotypes (Figure 7). Two ancestral lineages are noted (L1, L2). L1 subsequently evolved to the two indicated haplotypes, both of which have the increased expression phenotype. L2 underwent a more complex evolution resulting in two major lineages, L2(1) and L2(2). The former consists of two haplotypes both with the reference expression phenotype. L2(2) segregates into a single, distinct, increased-expression haplotype, and a three-haplotype clade consisting of two reference-expression haplotypes and one increased-expression haplotype. This latter haplotype (IV-1) differs from its nearest neighbor (IV-2) by one additional C in a poly-C tract in the 3′UTR. This may represent an ancestral replication error along this repetitive sequence of up to 14 C's. While there appears to be some consistency with the tree and expression phenotype, there was no readily discernible pattern with the dual phenotypic groups A, B and C of Figure 6. And furthermore, the increased agonist-promoted downregulation haplotypes I-1 and IV-3 are not in the same lineages (Figure 7) and differ by 12 SNPs (Table 1). Taken together, this may imply that *ADRB2* evolution was driven by β_2_AR expression rather than downregulation or the dual phenotype. This interpretation is consistent with most β_2_AR in the body being activated by circulating epinephrine, which shows variability in plasma concentration [14] but rarely reaches the high concentrations necessary for downregulation. Nevertheless, the high concentrations of β-agonist achieved during pharmacologic therapy do promote β_2_AR downregulation in lung cells [15], so this component of the dual phenotype should be considered in grouping haplotypes for potential clinical associations with therapeutic response.

In conclusion, we have utilized the most common *ADRB2* haplotypes (allele frequency of 0.05 or greater) to ascertain expression and agonist-promoted downregulation. The full intronless gene was cloned into a promoterless expression vector, so that phenotypes could be manifested by polymorphisms in the promoter, 5′UTR, coding, or 3′UTR regions. The great majority of association studies in asthma (and other diseases) which have considered the *ADRB2* as a candidate gene for risk, severity or other clinical phenotype, or as a pharmacogenomic locus, have utilized one or both of the nonsynonymous coding polymorphisms. The results of these association studies range from highly significant associations [16] to the lack of any association [17]. While the study designs are virtually always different, which could explain these inconsistencies, we have been concerned that these limited polymorphisms do not provide a sufficient density of genetic information to discriminate phenotypes. Indeed, there are known pairs of polymorphisms with low linkage-disequilibrium within *ADRB2* [9]. To begin to coalesce the >26 polymorphisms in *ADRB2* with cellular phenotypes, in the specific combinations that are observed in the population, we constructed the eight common haplotypes. We note that there are little or no “spare β_2_AR” in airway smooth muscle in relation to relaxation [1], so the ∼20–40% lower expression observed between some haplotypes could be related to clinically significantly lower bronchodilatory responses to β-agonist. A similar close relationship between receptor expression and function is present in cardiomyocytes where the β_2_AR subtype increases inotropy and acts to inhibit certain apoptosis events in progressive heart failure when catecholamine levels are elevated [18]. Thus non-selective β-blocker efficacy could be affected by β_2_AR haplotype. The other phenotype examined, receptor downregulation, is also relevant to therapeutic efficacy since this represents a major mechanism of tachyphylaxis during chronic agonist exposure. Association studies using full β_2_AR haplotypes with β-agonists in obstructive lung disease, or β-blockers in heart failure, have not been carried out to date. Given the phenotypes that we have defined, it would appear appropriate to carry out such studies with the haplotypes, or groups of haplotypes with similar cellular phenotypes. Such an approach may improve the predictivenesss of *ADRB2* variation with relevant clinical phenotypes and resolve discrepancies between studies.

## Methods

### Expression Vectors

The expression vector pcDNA 3.1(+) (Invitrogen, Carlsbad, CA, USA) was mutated to remove the CMV promoter by sequential digestion with MluI and NheI followed by blunt-end ligation. This modified promoterless vector is denoted pcDNA 3.1(+)/ΔCMV and the full *ADRB2* haplotypes (with the *ADRB2* promoter) were inserted into the Hind III/Xba1 sites. To generate the eight *ADRB2* haplotypes, the bacterial artificial chromosome RP11-44B19 was used as the template to amplify *ADRB2* (the haplotype IV-1 form). Several rounds of site-directed mutagenesis by methods previously described [19] were utilized to generate mutations at the 26 variable sites, so as to construct the eight haplotypes shown in Table 1. Full-length sequencing of the final construct was performed to verify the nucleotide changes and the integrity of the *ADRB2*.

### Cell Culture and Transfections

COS-7 cells (obtained from American Type Culture Collection) were grown in Dulbecco's modified Eagle's medium with 10% fetal calf serum, 100 units/ml penicillin and 100 µg/ml streptomycin and for all conditions were maintained at 37° in a 95% air 5% CO2 environment. Transfections were carried out using methods previously described [20]. Briefly, 5.0 µg haplotype construct with 15 µl Lipofectamine 2000 (Invitrogen) were added to 10^7^ cells and incubated for 6 hours. Then, fresh media was added and the media changed the next day. Eighteen hours after transfection the cells were treated with vehicle (0.1 mM ascorbic acid, representing baseline) or the β-agonist isoproterenol in the media for 48 hours, with an exchange of media and fresh isoproterenol after 24 hours.

### Radioligand Binding and Western Blots

Attached cells were washed three times with PBS and then scraped in 5 mM Tris (pH 7.40) 2 mM EDTA at 4° and then centrifuged at 33,000× g for 15 min. Membranes were resuspended in 75 mM Tris (pH 7.40), 12 mM MgCl_2_, 2 mM EDTA and radioligand binding with ^125^I-CYP carried out in triplicate as described [21]. Co-incubations with propranolol (10 µM) were used to define non-specific binding. Reactions were terminated by dilution in cold buffer and bound radioligand separated from free radioligand by vacuum filtration over glass fiber filters. The filters were counted in a gamma counter, and specific binding calculated as total minus nonspecific binding normalized to protein and expressed as fmol/mg. For Western blots, 15 µg of protein was electrophoresed through 10% SDS-polyacrylamide gels and transferred to nitrocellulose membranes as described [19]. Membranes were incubated with antibodies to β_2_AR (1∶200 dilution, Santa Cruz) or Na^+^/K^+^-ATPase (1∶200 dilution, Santa Cruz) for 1 hour and processed using enhanced chemiluminescence (GE Healthcare).

### Quantitative RT-PCR

RNA was prepared using TRIZOL (Invitrogen) as described [22]. Reverse transcriptase reactions were carried out with Moloney murine leukemia virus reverse transcriptase (MultiScribe, Applied Biosystems, Foster City, CA, USA), 500 ng of extracted RNA, and random hexamer primers. Real time PCR was carried out with 2 µl of diluted reverse transcription reaction using methods previously described [12]. The TaqMan probe and primers sets were from Applied Biosystems for *ADRB2* (Hs00240532_s1), which provided an amplicon of 66 bp representing nucleotides 500–565 of the coding region. For the control gene, probe and primer sets for phosphoglycerate kinase 1 (*PGK2*) were utilized (Applied Biosystems 4326318E) which provides for an amplicon of 75 bp. Reactions were carried out in an Applied Biosystems 7300 RT-PCR system. Threshold cycle (Ct) values were obtained (∼15 for *ADRB2* and ∼23 for *PGK2*) and relative mRNA levels calculated using a 2^−ΔCt^ method [23].

### Phylogenetic modeling

A phylogenetic tree was constructed using methods similar to those that we have previously described [24], [25]. The unweighted pair group method with arithmetic mean was utilized with the maximum likelihood model to estimate evolutionary distance; 1,000 bootstrap sampling was performed.

### Statistical Analysis

Data are shown as mean ± standard error. Statistical comparisons were by ANOVA with post-hoc 2-way t-tests. Significance was considered when p<0.05.
